# RNA structure determination by solid-state NMR spectroscopy

**DOI:** 10.1038/ncomms8024

**Published:** 2015-05-11

**Authors:** Alexander Marchanka, Bernd Simon, Gerhard Althoff-Ospelt, Teresa Carlomagno

**Affiliations:** 1 Structural and Computational Biology Unit, European Molecular Biology Laboratory, Meyerhofstrasse 1, 69117 Heidelberg, Germany; 2 Bruker BioSpin, Silberstreifen 4, 76287 Rheinstetten, Germany; 3 Helmholtz Zentrum für Infektionsforschung, Inhoffenstrasse 7, 38124 Braunschweig, Germany

## Abstract

Knowledge of the RNA three-dimensional structure, either in isolation or as part of RNP complexes, is fundamental to understand the mechanism of numerous cellular processes. Because of its flexibility, RNA represents a challenge for crystallization, while the large size of cellular complexes brings solution-state NMR to its limits. Here, we demonstrate an alternative approach on the basis of solid-state NMR spectroscopy. We develop a suite of experiments and RNA labeling schemes and demonstrate for the first time that ssNMR can yield a RNA structure at high-resolution. This methodology allows structural analysis of segmentally labelled RNA stretches in high-molecular weight cellular machines—independent of their ability to crystallize— and opens the way to mechanistic studies of currently difficult-to-access RNA-protein assemblies.

## Abstract (web summary)

The determination of RNA structures within high-molecular weight protein-RNA complexes in non-crystalline state is technically challenging. Here, the authors describe a solid-state NMR protocol for the determination of RNA structures at high resolution.

## Graphical abstract

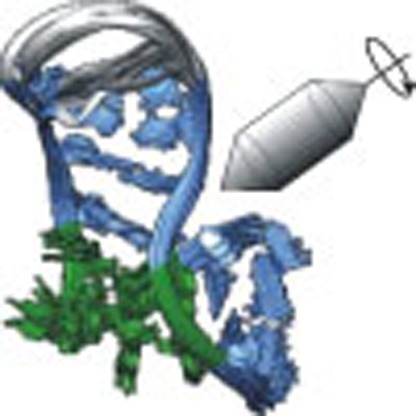

In gene expression regulation, stress response and pathogens infection, a multitude of non-coding RNAs and ribonucleoprotein complexes accomplish their function cycling through transient intermolecular contacts and related conformational changes. Taking influence on these processes requires a mechanistic understanding of the intermolecular interactions, which, in turn, necessitates structural information. Both naked RNAs and RNPs represent a challenge for structural biology. The conformational plasticity of the RNA restricts application of X-ray crystallography, while the high-molecular weight of the RNA (or RNP) of interest pushes solution-state NMR to its limits. Lately, solid-state NMR (ssNMR) spectroscopy, which is applicable to macromolecules of any size in non-crystalline form, has emerged as a powerful alternative to study the structure of amyloid fibrils^1^,^2^, membrane proteins^3^,^4^, and large protein–protein assemblies^5^. Despite these successes, ssNMR has been rarely applied to nucleic acids, and the methodology for RNA structure determination is still lacking^6^. Here, we present the first *de novo* structure determination of RNA by ssNMR, together with the experimental methods we developed for it. We demonstrate that RNA structure is accessible at high resolution by ssNMR using a few, easy to prepare, nucleotide-type selectively labeled samples. This methodology opens the way to the structure of RNA stretches in large RNA-protein assemblies, independent of their ability to crystallize, and thus to mechanistic studies of yet inaccessible cellular machines.

## Results

### The Box C/D RNA bound to L7Ae

**Figure 1:**
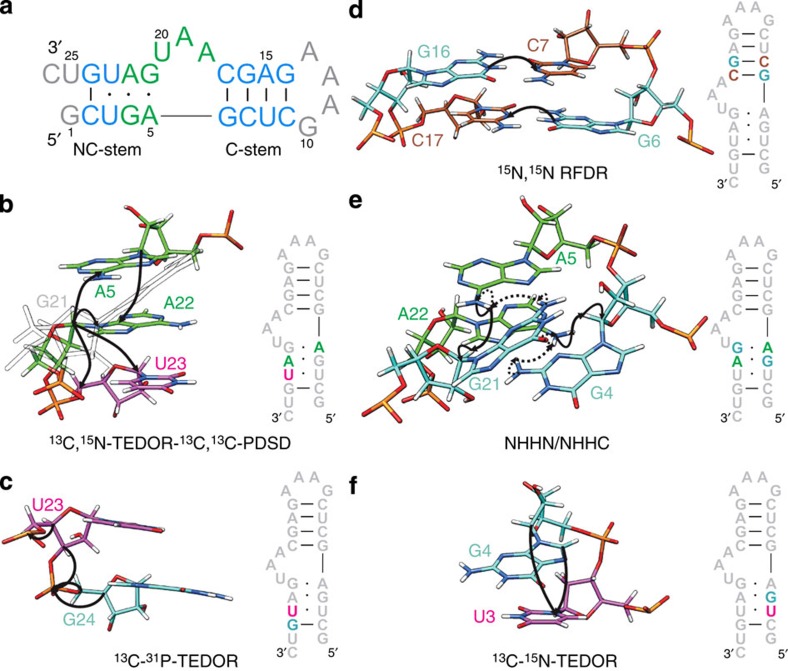
Sequence of the *Pf* Box C/D RNA and magnetization transfer schemes. (**a**) Sequence and secondary structure of the *Pf* Box C/D RNA. Helical regions, light blue; k-turn, green; loop and termini, grey. (**b**–**f**) Schematic representation of the magnetization transfer schemes used for resonance assignment and distance measurement, shown on nucleotides stretches highlighted in the sequence. A, green; G, cyan; C, sienna; U, magenta. (**b**) ^13^C,^15^N-TEDOR-^13^C,^13^C-PDSD, A,U^lab^-RNA. (**c**) ^13^C, ^31^P-TEDOR, G,U^lab^-RNA. (**d**) ^15^N,^15^N-RFDR, G,C^lab^-RNA. (**e**) NHHN (dotted) and NHHC (solid), A,G^lab^-RNA. (**f**) ^13^C, ^15^N-TEDOR, (G-^13^C,U-^15^N)^lab^-RNA.

In this study, we solve the structure of the 26mer Box C/D RNA from *Pyrococcus Furiosus* (*Pf*) as part of the complex with the protein L7Ae (Fig. 1a and Supplementary Fig. 1). ^13^C and ^15^N line widths of 0.4–0.5 and 0.6–0.8 p.p.m., respectively, allow determining the structure by ssNMR data with a precision of 0.8 Å. We choose to study this RNA for the following reasons. First, we were unable to crystallize the L7Ae-Box C/D RNA complex with the RNA sequence of Fig. 1a, despite the existence of crystallographic structures of homologues^7^,^8^. This demonstrates that the crystallization of RNA-protein complexes can be unexpectedly challenging, in dependence of the RNA sequence. Second, the RNA of Fig. 1a contains the conserved Box C and Box D sequences, which build the so-called k-turn motif. The geometry of the k-turn is measured by an angle *φ*^9^, which is variable in the free RNA and depends on the concentration of magnesium^10^,^11^. Upon protein binding, the k-turn parameter *φ* adopts a value close to 23° for all k-turn motifs investigated to date, independent of the experimental method, the exact sequence of the RNA or the species it belongs to ref. 12. The conservation of this structural motif offers the opportunity to verify the accuracy of the structure obtained by ssNMR, beyond the differences to crystallographic reference structures expected as a consequence of packing forces and RNA-RNA contacts in the crystals.

Resonance assignment and measurement of distance restraints are the key steps in structure determination by ssNMR. In contrast to proteins, where homonuclear ^13^C,^13^C correlations are sufficient for resonance assignment, the poor chemical shift dispersion of ribose resonances in RNA requires additional heteronuclear editing. We find that three-dimensional pulse schemes yield low signal-to-noise within our experimental set-up, while the quality of two-dimensional spectra allows for both assignment and quantification of cross-peaks. Therefore, our strategy does mainly without three-dimensional experiments and resolves spectral overlaps by selective labeling. To make the method accessible to a broad community, we abstain from using atom-selective labelling and employ only RNAs that can be produced with commercially available building blocks by *in vitro* transcription. In this study, we designed eight combinations of double-nucleotide-type selective-labelled RNAs (Supplementary Fig. 1), to accomplish both resonance assignment and measurement of structural parameters.

### Sequence-specific assignment

First, we assigned the spin-systems of individual nucleotides in a non-site-specific manner^13^. For the 26mer Box C/D RNA, we found six adenosines, seven guanosines, three cytosines and four uridines spin-systems. Analysis of canonical coordinates of ribose shifts^14^ (Supplementary Fig. 2) suggests that two adenosines, three guanosines and one uridine are not located in regular A-form helices. This allowed us to attribute the uridine spin-system to U20, which was used as starting point for sequential assignment.

**Figure 2:**
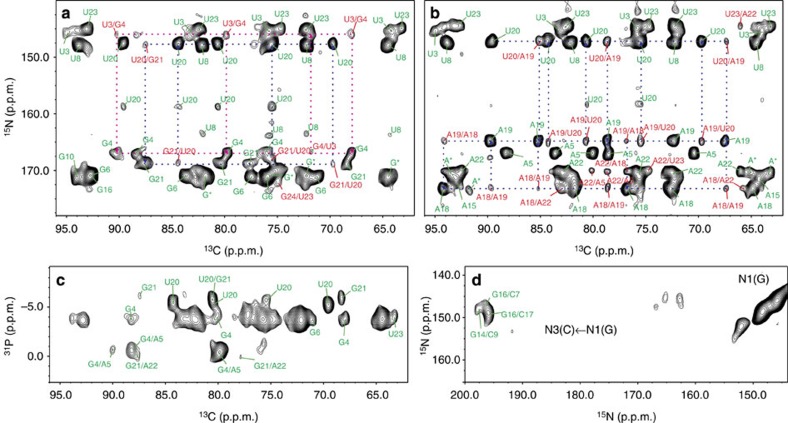
ssNMR spectra for the sequential assignment and measurement of structural restraints. (**a**–**b)** Ribose region of 2D ^13^C,^15^N-TEDOR-^13^C,^13^C-PDSD spectra of (**a**), G,U^lab^-RNA and (**b**) A,U^lab^-RNA (mixing time, 700 ms). Intra- and inter-nucleotide correlations are labeled in green and red, respectively. Selected sequential correlations are shown. Partially overlapped guanosines G10, G14, G16 are labeled as G*; non-site-specifically assigned adenosines in the tetra-loop (A11–A13) are labeled as A*. (**c**) 2D ^13^C,^31^P-TEDOR spectrum of G,U^lab^-RNA. (**d**) 2D ^15^N,^15^N-RFDR spectrum showing the G-N1/C-N3 correlations for G:C base pairs.

Seventeen out of the 26 nucleotides were assigned site-specifically using correlations between the C1' or C6/C8 atoms of nucleotide *i* to the carbons of neighbouring nucleotides *i*±*1*, as well as nucleotides of the opposite strand (Fig. 1b, Supplementary Table 1). To improve resolution, before the ^13^C,^13^C transfer, the magnetization of C1' or C6/C8 was correlated to the respective N1/N9 via TEDOR (Transferred-Echo-DOuble-Resonance)^15^, yielding a two-dimensional (2D) ^15^N,^13^C correlation (Fig. 2a,b, Methods). This allows clear distinction of purine and pyrimidines in double-nucleotide-type selective-labelled samples (for example, G,U^lab^- or A,U^lab^-RNAs). For the long-range carbon–carbon transfer, we tested different mixing sequences and finally settled on the PDSD (Proton-Driven-Spin-Diffusion) scheme due to its superior sensitivity^16^.

^13^C,^15^N-TEDOR-^13^C,^13^C-PDSD (Supplementary Fig. 3a) was applied with a mixing time of 700 ms to six selective-labeled RNAs (Supplementary Fig. 1) and yielded several inter-nucleotide contacts up to a distance of 9–10 Å (Fig. 2a,b, Supplementary Figs 4 and 5). As an example of sequential assignment, G,U^lab^- and A,U^lab^-RNAs yielded multiple correlations between the C1',C6 of U20 and both a guanosine and an adenosine spin-system; the latter correlates further with another adenosine of the A,U^lab^-RNA. These cross-peaks are compatible with either an AAUG or a GUAA stretch, and identify unambiguously the spin-system A18-A19-U20-G21. This strategy yielded sequential assignment of 17 out of 19 nucleotides in structured regions, excluding the tetra-loop and the terminal ends. The remaining two nucleotides (G14, A15) were assigned by substitution of the tetra-loop sequence GAAA with UUCG. This alleviated the overlap of G14 and A15 with the resonances of the GAAA loop, allowing their assignment, as well as the identification of G10 and of two adenosines of the A11–A13 stretch. The poor intensity of the GAAA tetra-loop resonances is indicative of conformational heterogeneity; likewise, the terminal G1, U25 and C26 spin-systems are not visible in any of the spectra and were not considered in the structure calculation.

Next, we tested the performance of a ^13^C,^31^P correlation, which, with a TEDOR mixing time of 3.2 ms, should provide sequential C2'_i_/P_i+1_ and C3'_i_/P_i+1_ contacts. The mixing time was optimized for sensitivity of transfer over two to three bonds, ranging up to 4–5 Å distance (Fig. 1c). As expected, the ^31^P resonances are poorly resolved in helices and the spectrum provided information only for non A-form structural elements (Fig. 2c, Supplementary Fig. 6a,b).

Finally, we could sequentially assign >90% of all carbon resonances of the *Pf* Box C/D RNA in the stretches 2–10 and 14–24 (81% for both carbons and nitrogens).

### Structural determination of the RNA by ssNMR

The determination of RNA secondary structure requires the identification of base pairs. To this end, we used a ^15^N,^15^N through-space correlation (RFDR, Radio-Frequency-Driven-Recoupling)^17^,^18^ to reveal the spatial proximity of either A-N1 and U-N3 or G-N1 and C-N3 in Watson–Crick base pairs (Fig. 1d). The presence of three G:C base pairs (Fig. 2d) defined the C-stem. The G24:C2 base pair was not found due to the absence of the G24-N1 resonance in intra-nucleotide correlations, probably as a consequence of conformational heterogeneity at the helix ends.

Secondary structure prediction suggests one U·U and two A·G base pairs (Fig. 1a). Initially, to verify the presence and determine the topology of these non-canonical base pairs, we measured NHHN spectra^19^,^20^; in this experiment, magnetization is transferred between close-by ^15^N nuclei exploiting the spatial proximity of their attached protons (Fig. 1e). This strategy failed, due to severe overlap of the involved nitrogen resonances. Next, we recorded NHHC spectra (Fig. 1e) on three selectively labelled RNA samples (Supplementary Fig. 7); (G,U)^lab^-RNA yielded weak N2_G21_/C1'_G4_ correlations, while (A,G)^lab^-RNA yielded strong N6_A22_/C1'_G4_ and N6_A5_/C1'_G21_ signals. The last two correlations were also detected in a ^13^C-band-selective, ^15^N-TEDOR spectrum (Supplementary Fig. 6c). This pattern of cross-peaks, together with the *anti* conformation of the glycosidic angle *χ* for all four G and A nucleotides (*vide infra*), is exclusively compatible with two N7-amino, N3-imino base pairs, which are typical of k-turn motifs.

The U3·U23 base pair might be detected from the proximity of the two H3 atoms in a NHHN correlation. In our case, the chemical shift difference of only 1 p.p.m. between the U3-N3 and U23-N3 hindered the resolution of the weak cross-peak from the intense diagonal. Therefore, we resorted to the analysis of chemical shifts (CS), as indicators of secondary structure. The CS of U23-C2 (151 p.p.m.) and both U3- and U23-C4 (165.6 and 167.6 p.p.m., respectively) deviate from the values of non-stacked disordered nucleotides (154.0 and 168.5 p.p.m.) as well as from the values of A-form helices (152.9 and 169.2 p.p.m.; ref. 21). The low CS of U23-C2 and U3-C4 indicate stacking on both sides, while for U3-C2 and U23-C4 the up-field shift induced by stacking is compensated by the down-field shift of carbonyl acceptors of H-bonds. All in all, CS analysis predicts that U3 and U23 form a U3·U23 2-carbonyl-N3, 4-carbonyl-N3 base pair^22^.

### Distance restraints

Next, we obtained distance restraints from four different correlation experiments: ^13^C,^15^N-TEDOR-^13^C,^13^C-PDSD recorded at multiple mixing times provided carbon–carbon distances; ^13^C,^31^P-TEDOR and ^13^C-band-selective, ^15^N-TEDOR yielded a few carbon–phosphorus (17) and carbon–nitrogen (6) distances, respectively; CHHC and NHHC experiments yielded distances between protons (Supplementary Table 2). In this context, we proved the applicability of more sophisticated and selective transfer schemes, such as PAR (Proton-Assisted-Recoupling) and PAIN (Proton-Assisted-Insensitive-Nuclei)^23^,^24^. However, the sensitivity of these experiments remained too low, especially in combination with heteronuclear filtering.

The mixing sequence PDSD does not permit the quantitative measurement of distance restraints^25^,^26^; however, when recorded at multiple mixing times, it provided information on (C1',C8/C6)_*i*_–(C_*x*_)_*j*_ distance ranges. A total of 91 inter-nucleotide cross-peaks were obtained from the ^13^C,^15^N-TEDOR-^13^C,^13^C-PDSD experiments, which were all incorporated in structure calculations, in addition to 46 intra-nucleotide restraints over ≥3 bonds (Supplementary Table 2).

Next, we attempted to obtain base–base C_*i*_–N_*i*±1_ cross-peaks through a ^13^C-band-selective,^15^N-TEDOR experiment^15^ recorded for samples with ^13^C-labelling of one nucleotide type and ^15^N-labelling of another nucleotide type (Fig. 1f). Our efforts were unsuccessful, due to low signal-to-noise. However, when recording a (^13^C1',^13^C4')-band-selective,^15^N-TEDOR, we obtained six inter-nucleotide cross-peaks from both the k-turn and helical regions (Supplementary Fig. 6c,d and Supplementary Table 2).

Finally, 2D NHHC and CHHC spectra^19^,^27^ yielded 17 and 10 inter-nucleotide contacts (Supplementary Figs 7 and 8), respectively, in addition to 21 intra-nucleotide correlations over ≥3 bonds (Supplementary Table 2).

In addition to distance restraints, we obtained dihedral angles from analysis of ribose chemical shifts (Methods and Supplementary Fig. 2) and from CHHC experiments at short mixing times. Similarly to solution-state NMR, the *χ* angle was restrained to *syn* in the presence of a strong C1'-C8/C6 cross-peak (short H1'-H8/H6 distance) and to *anti* in the other cases. Only A19 displayed a *χ* angle in the *syn* conformation, in agreement with other k-turn RNA structures^11^.

### Structure calculations

**Figure 3:**
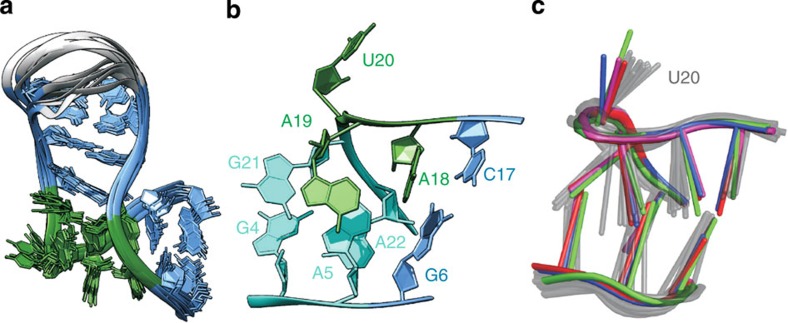
ssNMR structure of the *Pf* Box C/D RNA. (**a**) Overlay of the 10 lowest energy structures of the *Pf* Box C/D RNA in complex with L7Ae from ssNMR data. Terminal nucleotides 1 and 25–26 are not shown. Colour code as in Fig. 1a. (**b**) k-turn of the *Pf* Box C/D RNA, showing the characteristic geometry. Internal loop, green; NC stem, cyan, C stem, light blue. (**c**) Comparison of the k-turn geometry of the *Pf* Box C/D RNA obtained by ssNMR (10 lowest energy structures, gray) with that of the crystallographic structure of the *Af* Box C/D RNA (PDB code 1RLG)^7^, red; *Pf* Box C/D RNA (PDB code 3NMU)^8^, blue; *Af* Box C/D RNA (PDB code 4BW0)^29^, green; *Ss* Box C/D RNA (PDB code 3PLA)^30^, magenta.

Distance and dihedral angle restraints, as well as base pair restraints were used in ARIA^28^ to calculate the structure of the Box C/D RNA from ssNMR data. Out of 300 calculated structures, the first 60 converged to a well-defined minimum with precision of 0.9 Å (root-mean-square-deviation (r.m.s.d.) of all heavy atoms of nucleotides 2–9 and 14–24 of the first 20 structures; Fig. 3a and Table 1). The distances derived from the ^13^C,^15^N-TEDOR-^13^C,^13^C-PDSD spectra had the highest impact on the precision of the structure, followed by those derived from the CHHC and NHHC experiments (Supplementary Fig. 9). As for structural calculation from solution-state NMR data, the definition of the RNA secondary structure (topology of base pairs) was essential. The stem regions were defined by eight distance and nine angular restraints per residue, while the geometry of the k-turn required 21 distances per nucleotide. The structure determination method was validated by removal of random fractions of restraints. The structures bundles were consistent upon random removal of up to 20% of the total restraints.

## Discussion

The 26mer Box C/D RNA used in this study does not crystallize in complex with L7Ae; however, the crystallographic structure of two orthologous complexes from *Archaeoglobus fulgidus*^7^,^29^ (*Af*, PDB code 1RLG and 4BW0), one orthologous complex from *Solpholobus solfataricus*^30^ (*Ss*, PDB code 3PLA) and another L7Ae-Box C/D RNA from *Pf* with a different RNA sequence^8^ (PDB code 3NMU) let us evaluate the accuracy of the ssNMR structure in the critical k-turn region (Fig. 3b,c). The *φ* angle of 23° that defines the k-turn geometry of the ssNMR structure is in very good agreement with the *φ* angles of the reference structures (1RLG, 23°; 4BW0, 22°; 3PLA, 24°; 3NMU, 24°).

Next, we analysed the backbone and glycoside torsion angles of our structures bundle and compared them with the corresponding torsion angles of the four reference structures (Supplementary Fig. 10). We choose to compare torsion angles rather than r.m.s.d. values to better visualize the variability of both the crystallographic structures and our bundle at each nucleotide position. The *δ* torsion describes the ribose pucker and is defined by the chemical shift analysis of Supplementary Fig. 2. The values fit nicely to those of the reference structures, with the exception of 5, 19 and 20 of 1RLG, which adopt the C3'-endo conformation. Our NMR data indicate that the conformation of these riboses is C2'-endo, in agreement with the other three crystallographic structures. Similarly, the *ɛ* and *ζ* angles of the same nucleotides of 1RLG deviate from the values of both our structures bundle and the other three crystallographic structures. The *β*, *ɛ* and *ζ* torsion angles are not directly determined by any NMR parameter, but rather restrained loosely by data base values (see Methods), ^31^P–^13^C and ^13^C–^13^C distances. Nevertheless, the distribution of these angles in the ssNMR bundle is quite narrow and in good agreement with the reference structures. The *α* and *γ* torsion angles are the least well defined by the NMR distance restrains in the stretch 18–21 of the Box C sequence. Interestingly, high variability is observed for these torsion angles among the four reference structures as well, indicating that the k-turn geometry is tolerant to different values (Supplementary Fig. 10). The only clear discrepancy between the ssNMR structures bundle and the four references structures is observed for A5-*α*,*γ*. The A5-^31^P chemical shift value (Supplementary Table 1) does not allow to restrict the A5-*α* to the gauche±conformations^31^, as observed in the four reference structures. However, despite this local difference, the k-turn geometry of the ssNMR bundle agrees very well with that of the reference conformations, with an average backbone r.m.s.d. for k-turn nucleotides 4–6 and 17–22 of 1.3 Å to the four crystallographic structures (Fig. 3c). Finally, the glycosidic torsion angles *χ* are determined from the intensities of the C1'-C8/C6 cross-peaks and nicely agree with those of the reference structures.

In summary, we demonstrate that the structure of RNA is accessible by ssNMR with excellent precision and accuracy, despite the difficulties caused by broad line widths and resonance overlap. We present a straightforward, manageable strategy that uses easy-to-produce nucleotide-type selective-labelled RNAs and sensitive magnetization transfer schemes. Our results make the folding of short RNAs and selectively labelled RNA stretches, as well as their interaction with proteins, accessible at high resolution in the context of large RNAs and RNP particles. We anticipate that our method will have a considerable impact in various fields of RNA processing and small RNA regulation (siRNA, miRNA, piRNA), where the dynamic nature of the molecular complexes represents an obstacle to crystallization.

## Methods

### Sample preparation

The L7Ae–Box C/D RNA complex was assembled from protein and RNA in 1:1 ratio and purified by size exclusion chromatography. L7Ae was expressed in *Escherichia coli* (LB medium) and purified over a Ni-Nta column. Nucleotide-type ^13^C, ^15^N selective-labeled Box C/D RNA was prepared by *in vitro* transcription with T7 polymerase produced in house. Labeling patterns of the RNA were obtained using NTP mixtures where only one or two nucleotide types were either ^15^N or ^13^C, or double ^13^C,^15^N labeled.

Sequential resonance assignment and measurement of structural restraints used eight samples with different labeling patterns. Six samples consisted of double ^13^C,^15^N nucleotide-type selective-labelled RNAs: A,C^lab^-RNA, A,G^lab^-RNA, A,U^lab^-RNA, C,U^lab^-RNA, G,C^lab^-RNA and G,U^lab^-RNA (Supplementary Fig. 1a–f); two samples contained single ^13^C or ^15^N labelled nucleotide pairs: (G-^13^C, A-^15^N)^lab^-RNA and (G-^13^C, U-^15^N)^lab^-RNA (Supplementary Fig. 1g,h). Next to these RNAs, an additional RNA construct was used to facilitate assignment, where the GAAA tetra-loop is substituted with the UUCG tetra-loop.

The L7Ae–Box C/D RNA complex was concentrated to 20 mg ml^−1^ in buffer containing 25 mM HEPES and 120 mM sodium chloride at pH 7.5, and subsequently mixed with equal amount of precipitation solution (100 mM sodium acetate, 30% PEG 400 in 100 mM HEPES, pH 7.5), as reported previously^13^,^32^,^33^. The sample was micro-crystallized by slow precipitation using a SpeedVac concentrator at room temperature for ∼2.5 h. The complex precipitated at half volume. The precipitate was packed in the ssNMR rotor by centrifugation. The final sample contained ∼4 mg of RNA and 6 mg of L7Ae.

### NMR spectroscopy

Solid-state NMR experiments were performed on a 700 MHz SB Bruker Avance III spectrometer equipped with 3.2 mm MAS ^1^H/^13^C/^15^N probehead. ^13^C,^31^P TEDOR experiments were acquired at 600 MHz with a WB Bruker Avance III spectrometer equipped with a tunable ^1^H/X/Y probehead at Bruker Biospin in Rheinstetten. The temperature of all experiments was 260 K. ^13^C,^15^N-TEDOR-^13^C,^13^C-PDSD, ^13^C,^31^P-TEDOR, ^13^C,^15^N-TEDOR and ^15^N,^15^N-RFDR experiments were performed at 16 kHz MAS, while proton diffusion-based CHHC and NHHC experiments were performed at 13 kHz MAS.

### ^13^C,^15^N-TEDOR-^13^C,^13^C-PDSD

In the ^13^C,^15^N-TEDOR-^13^C,^13^C-PDSD experiment (Supplementary Fig. 3a) ^13^C magnetization was prepared by standard ^1^H–^13^C cross polarization (mixing time, 200 μs). The ^13^C–^15^N dipolar coupling was reintroduced in a short TEDOR mixing time (1.5–2 ms), during which magnetization was transferred to nearby ^15^N nuclei, and then, after *t*_1_, back to the ^13^C. In *t*_1_, we recorded the frequency of nitrogens close to carbons, as for example that of N1/N9 directly bound to C1' and C8/C6. The following, long ^13^C,^13^C-PDSD step (mixing time, 200–700 ms) transferred the ^15^N-chemical shift labelled ^13^C magnetization to nearby carbons. Finally, ^13^C magnetization was detected during *t*_2_. The ambiguity on the carbon from which the magnetization originates in the PDSD step, either C1' or C8/C6, was lifted in three-dimensional experiments, where the ^13^C frequency was recorded before the PDSD mixing. Alternatively, we evaluated the efficiency of the ^15^N1/N9-^13^C transfer, which in several instances was found to be better towards the C1' than towards C8/C6. As a third alternative, a ^13^C-band-selective-TEDOR transfer, with selectivity either on C1' or on C6/C8, can be used to resolve the ambiguity. Cross-peaks were evaluated and translated into distance restraints. Distance ranges (*d*) were applied for inter-nucleotide restraints as 3.5<*d*<9 Å, according to several previous studies^34^,^35^,^36^; the ranges for intra-nucleotide base–ribose restraints, 3<*d*<6 Å, and intra-nucleotide ribose–ribose restraints, 3<*d*<4 Å, were determined from the nucleotides' geometry.

### ^13^C,^31^P-TEDOR

In the ^13^C,^31^P-TEDOR experiment (Supplementary Fig. 3b), after initial preparation of ^13^C magnetization, the ^13^C,^31^P dipolar coupling was re-introduced in a TEDOR mixing time of 3.2 ms; the frequency of ^31^P was monitored in *t*_1_, while ^13^C magnetization was detected during *t*_2_. Optionally, a short ^13^C,^13^C-PDSD step (50–100 ms) can be applied after TEDOR to transfer the ^13^C magnetization to further carbon spins, such as C1'. This experiment was useful to identify the ribose spin systems through the better-resolved C1' chemical shift. ^13^C,^31^P-TEDOR spectra were recorded for A^lab^-RNA and G,U^lab^-RNA; due to the limited signal-to-noise, only one TEDOR mixing time was recorded (3.2 ms). The spectra yielded 17 non-trivial restraints, which were classified as 3<*d*<5 Å, as appropriate for a mixing time of 3.2 ms.

### ^13^C-band-selective, ^15^N-TEDOR

In the ^13^C-band-selective,^15^N-TEDOR experiment^9^ (Supplementary Fig. 3c), after initial preparation of ^13^C magnetization, the ^13^C–^31^N dipolar coupling was reintroduced in a TEDOR mixing time of 6–15 ms with band-selective ^13^C inversion pulses; the long mixing allows transferring magnetization between carbons and nitrogens as far as 5–6 Å. The ^15^N and ^13^C frequencies were recorded during *t*_1_ and *t*_2_, respectively. With a ^13^C,^15^N-TEDOR that was selective for C1' and C4', we obtained four G-C1',C4'/A-N6,N9 cross-peaks from the (G-^13^C,A-^15^N)^lab^-RNA and two G-C1'/U-N1,N3 cross-peaks from the (G-^13^C,U-^15^N)^lab^-RNA. Also in this case, we did not acquire multiple TEDOR mixing times, due to limited signal-to-noise. Distance ranges 3<*d*<5 Å and 3<*d*<7 Å were attributed to the strong and weak peaks, respectively, at a mixing time of 12 ms.

### ^15^N,^15^N-RFDR

In the ^15^N,^15^N-RFDR experiment (Supplementary Fig. 3d), ^15^N magnetization was prepared through a 300 μs cross-polarization step and its frequency was recorded during *t*_1_; subsequently, the magnetization was transferred to nearby nitrogen atoms via an RFDR mixing step of 20 ms and finally detected during *t*_2_.

### CHHC and NHHC

In the CHHC and NHHC proton spin diffusion-based experiments (Supplementary Fig. 3e,f, respectively), ^13^C or ^15^N magnetization was prepared through a short cross-polarization mixing time of 100–200 μs, followed by *t*_1_ evolution on either ^13^C (CHHC) or ^15^N (NHHC). Next, the magnetization was transferred back to protons, from where, after a short proton mixing of 100–200 μs, it was transferred to nearby carbons with a 100-μs cross polarization step; finally the frequency of ^13^C was recorded in *t*_2_. Inter-proton distances 2<*d*<4 and 2<*d*<5 Å were attributed to the strong and weak signals, respectively, following previous studies^19^,^27^.

In all the experiments, protons were decoupled in the indirect and direct acquisition times using high-power SPINAL-64 (ref. 37) decoupling at 85–95 kHz. Chemical shifts were referenced as described by Morcombe and Zilm^38^. The spectra were processed with NMRPipe^39^ and visualized with NMRviewJ^40^.

### Structural calculation protocol

Structures were calculated using the Aria 1.2/CNS 1.1 set-up^28^,^41^ following a similar protocol as for structural calculations of RNA by solution-state NMR data^11^,^42^,^43^. Both canonical and non-canonical base-pairs were incorporated in the structure calculation as distance restraints. Planarity was enforced through weak planarity restraints (5 kcal mol^−1^ Å^−2^) for canonical base pairs and non-canonical base pair U3·U23. Flexible planarity was introduced for the base pairs A5·G21 and G4·A22 by defining the plane that involves one atom of the acceptor and four atoms of the donor base to allow for propeller twist and tilt, as described in ref. 44.

The ribose conformation was restrained through the analysis of ribose chemical shifts^14^ (Supplementary Fig. 2). The riboses of nucleotides G4, A5, A19, U20, G21 were given an S-type conformation, while the remaining nucleotides, except for G6 and A11-A13, were restrained to the N-type conformation. The dihedral angles *α*, *β*, *ɛ* and *ζ* were restrained to the range typical for A-form helix (300°±30°, 180°±30°, −135°±30° and 300°±30°, respectively) for nucleotides 2, 6–9, 14–17, 24, which are involved in canonical base pairs; the *α*, *β*, *ɛ* and *ζ* angles of the remaining nucleotides were loosely restrained to the allowed ranges (180°±150°, 180°±110°, −125°±75° and 180°±150°, respectively). Dihedral angles *α* and *ζ* of nucleotides G4, G6, A18, U20 and G21 were additionally restrained to 0°±120° based on ^31^P chemical shifts^31^. The dihedral angle *γ* was restrained to the *gauche+* conformation for nucleotides involved in base pairs.

Three hundred structures were calculated in one iteration without the automated assignment or the distance calibration options of Aria 1.2 using an assigned distance list. Before minimization, we randomized all backbone dihedral angles. The minimization protocol used the force-field DNA-RNA-allatom-hj-opls.top and the following parameters in the four steps of simulated annealing (SA), together with the PROLSQ nonbonded parameters^43^: (i) the SA protocol started with a high-temperature torsion angle simulated annealing phase of 100,000 steps at 20,000 K (time step of 22.5 fs); (ii) this was followed by a torsion angle dynamic cooling phase from 20,000 to 1,000 K in 100,000 steps and by two cartesian dynamic cooling phases with a time step of 2.5 fs ((iii) from 2,000 to 1,000 K in 100,000 steps and (iv) from 1,000 to 50 K in 80,000 steps, respectively. Finally, 20 low energy structures were refined in water (TIP3P) with OPLS nonbonded parameters^45^. Standard ARIA force constants were used for the different restraint types (for example, distances—50 kcal mol^−1^, and dihedrals—200 kcal mol^−1^, in the final cooling step.)^28^.

The final structures were analysed using MolMol^46^ and Chimera^47^. Figures were prepared with Chimera.

### Analysis of the mutant Box C/D RNA with the UUCG tetra-loop

We measured two samples of the mutant Box C/D RNA containing the stable UUCG tetra-loop (UUCG-RNA) instead of the GAAA tetra-loop to aid and confirm the assignment. The 2D ^13^C,^15^N-TEDOR-^13^C,^13^C-PDSD of the (A,U)^lab^-UUCG-RNA allowed identifying two adenosines of the GAAA tetra-loop of the wild-type RNA, which disappear in the mutant spectrum. In addition, we could confirm the assignment of A15, which does not overlap with any other spin-system in the UUCG-RNA. The 2D ^13^C,^15^N-TEDOR-^13^C,^13^C-PDSD spectrum of G,C^lab^-UUCG-RNA allowed the assignment of G10, whose spin-system is not present in the spectrum of the mutant RNA. In addition, the resonances of G14 shift slightly in the mutant with respect to the wild-type RNA, due to the different structure of the UUCG tetra-loop. This fact confirmed the assignment of the G14 spin-system.

## Author contributions

A.M. produced samples, developed and performed both NMR experiments and structure calculations, interpreted the results and wrote the manuscript together with T.C. B.S. contributed to the development of pulse programs and structural calculation protocols. G.A. helped in the acquisition of NMR data. T.C. designed the project, contributed to the development of pulse programs and structural calculation protocols, interpreted the results and wrote the manuscript together with A.M. All the authors discussed the results reported in the manuscript.

## Additional information

**Accession codes:** The atomic coordinates of 10 lowest energy structures have been deposited in the Protein Data Bank under accession number 2N0R. The NMR chemical shifts have been deposited in the Biological Magnetic Resonance Data Bank, entry 25534.

**How to cite this article:** Marchanka, A. *et al*. RNA structure determination by solid-state NMR spectroscopy. *Nat. Commun*. 6:7024 doi: 10.1038/ncomms8024 (2015).

## Supplementary Material

Supplementary Information Supplementary Figures 1-10, Supplementary Tables 1-2 and Supplementary References

## Figures and Tables

**Table 1:** Structural statistics (20 structures out of 300 calculated, PDB code 2n0r).

NMR distance and dihedral constraints		
Distance restraints		
Total distance restraints	208	
Intra-residue	73	
Inter-residue	135	
Sequential (|i-j|=1)	96	
Non-sequential (|i-j|>1)	39	
Hydrogen bonds	34	
Total dihedral angle restraints	174	
Glycosidic angle *χ*	18	
Sugar pucker	54	
Backbone	102	
Based on A-form geometry	43	
		
**Structure statistics**	**In vacuum**	**Solvent refined**
		
Violations (mean and s.d.)		
Distance constraints (Å)	0.0043±0.0005	0.007±0.003
Dihedral angle constraints (°)	0.03±0.02	0.28±0.07
Max. distance constraint violation (Å)	0.05	0.24
Max. dihedral angle violation (°)	0.7	3.4
		
Deviations from idealized geometry		
Bond lengths (Å)	0.000182±0.00006	0.0023±0.0001
Bond angles (°)	0.461±0.002	0.64±0.02
Impropers (°)	0.319±0.001	0.41±0.02
		
Average pairwise r.m.s.d. (Å)		
All RNA heavy (2–9,14–24)	0.9±0.2	1.0±0.2
All RNA backbone (2–9,14–24)	0.8±0.2	1.0±0.2
Kink-turn backbone (4–6,17–22)	0.7±0.2	0.8±0.2
