# Supplementary material for: RNA structure determination by solid-state NMR spectroscopy
Source: Nat Commun. 2015 May 11;6:7024. doi: 10.1038/ncomms8024 (PMC4432599; doi:10.1038/ncomms8024)
Supplement: Supplementary Information — Supplementary Figures 1-10, Supplementary Tables 1-2 and Supplementary References [file ncomms8024-s1.pdf]

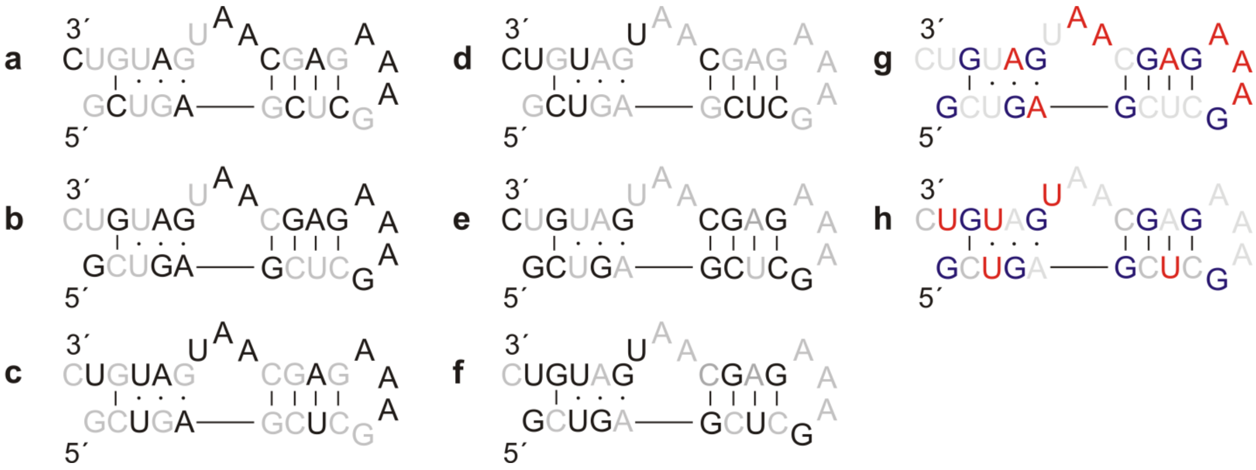

Supplementary Figure 1 | Overview of samples used for the structure determination of the *Pf* 26mer Box C/D RNA. a, A,C^{lab}-RNA, **b,** A,G^{lab}-RNA, **c,** A,U^{lab}-RNA, **d,** C,U^{lab}-RNA, **e,** G,C^{lab}-RNA, **f,** G,U^{lab}-RNA, **g,** (G-^{13}C/A-^{15}N)^{lab}-RNA, **h,** (G-^{13}C/U-^{15}N)^{lab}-RNA.

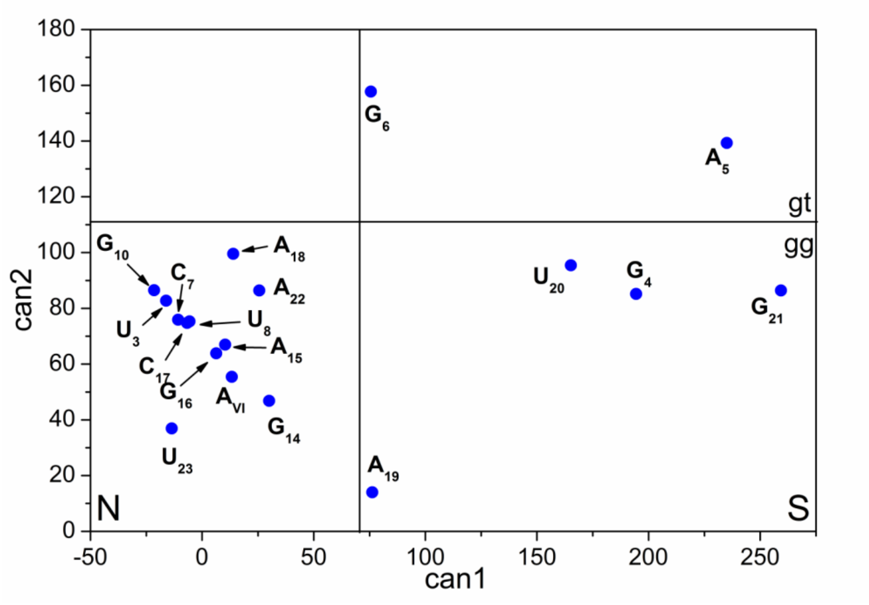

Supplementary Figure 2 | Canonical coordinates constructed from the ribose chemical shifts. S (south) and N (north) indicate the C2'-endo and the C3'-endo conformations of the ribose, respectively; gg and gt indicate the gauche/gauche and the gauche/trans conformations of the γ dihedral angle of the RNA backbone[1].

$can1 = -14.7\delta_{C1'} + 22.1\delta_{C2'} + 13.3\delta_{C3'} + 6.5\delta_{C4'} - 2.9\delta_{C5'} - 1595$

$can2 = 9.8\delta_{C1'} + 16.5\delta_{C2'} - 0.5\delta_{C3'} - 1.7\delta_{C4'} + 13.5\delta_{C5'} - 2781.$

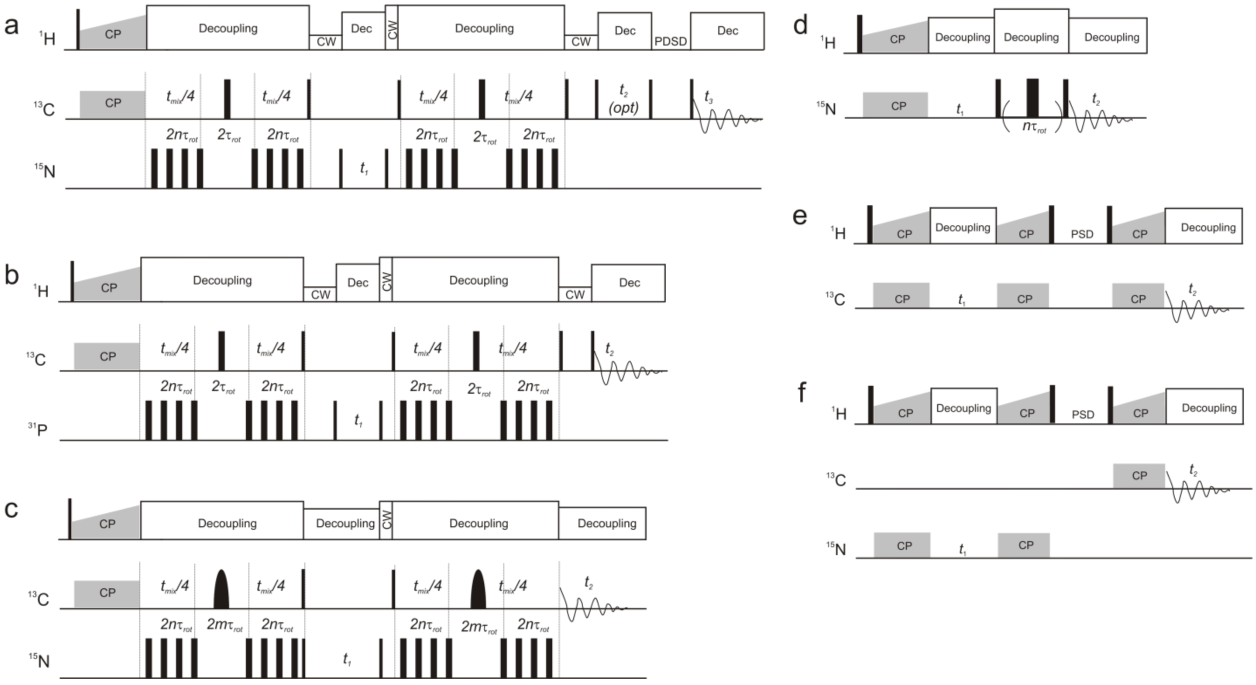

Supplementary Figure 3 | Pulse sequences for the experiments used in this study. Narrow and wide solid rectangles represent $\pi/2$ and π pulses, respectively. In all experiments the ^{1}H $\pi/2$ pulse width was 2.7-3.0 μs, while the ^{13}C and ^{15}N $\pi/2$ pulse widths were both 6 μs. **a**, ^{13}C,^{15}N-TEDOR-^{13}C,^{13}C-PDSD experiment. The TEDOR mixing time, t_{mix}, was 1.5-2 ms, the PDSD mixing time was 200-700 ms. **b,** ^{13}C,^{31}P-TEDOR[2] experiment. The ^{31}P $\pi/2$ pulse width was 6 μs; the sensitivity optimized TEDOR mixing time was 3.2 ms. **c,** ^{13}C-band-selective, ^{15}N-TEDOR experiment[2]. The width of the band-selective ^{13}C refocussing pulse was set to 8.5 rotor periods (531.25 μs), with the ^{13}C carrier placed at 90 ppm. **d,** ^{15}N,^{15}N-RFDR experiment[3]. The RDFR mixing time was 20 ms. **e** and **f**, CHHC and NHHC experiments[4,5]. The PSD (Proton Spin Diffusion) mixing time was 100-200 μs. CP, cross polarization; CW, continuous wave decoupling; Dec, decoupling; τ_{rot}, rotor period.

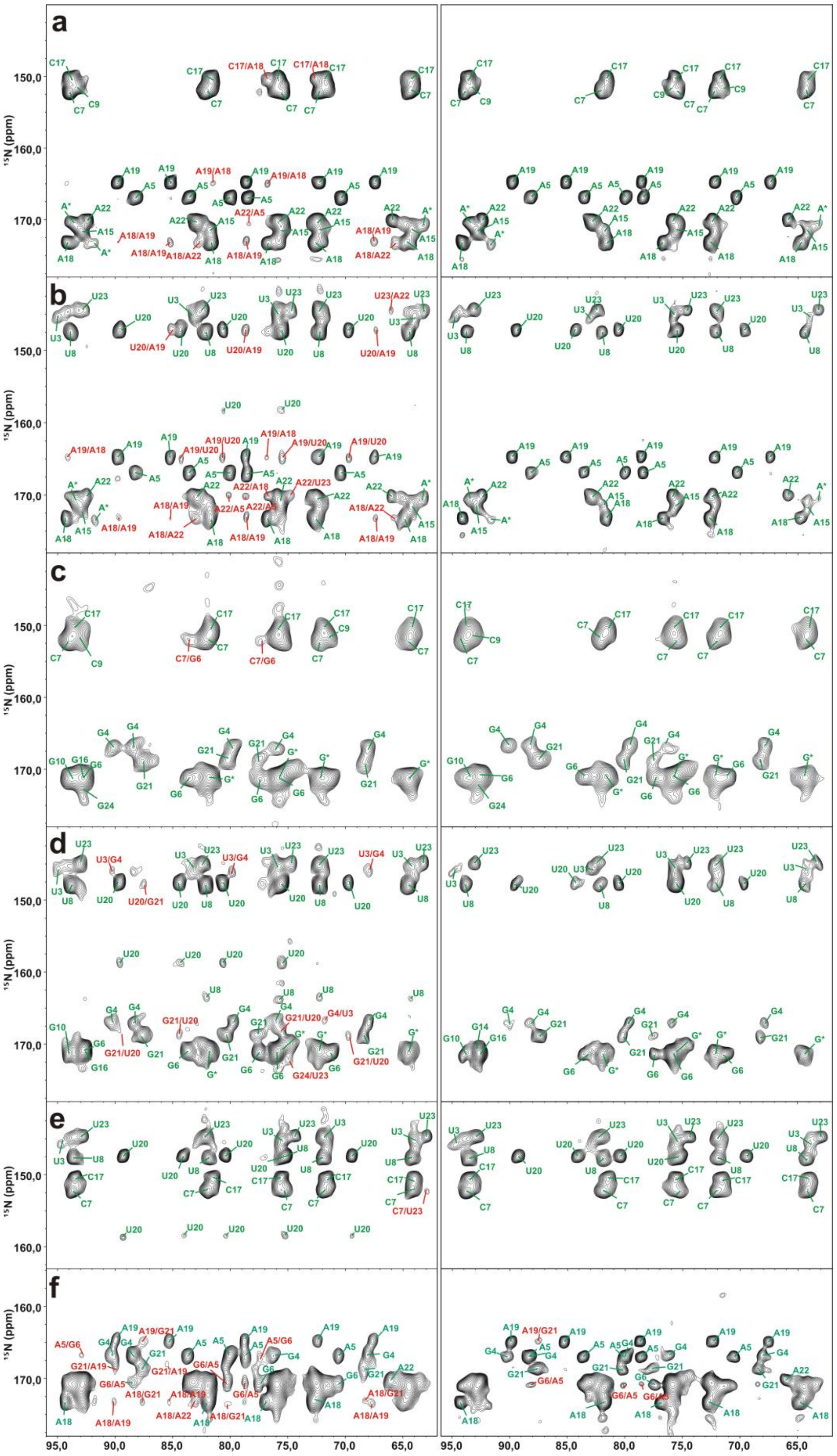

Supplementary Figure 4 | Ribose regions of 2D ^{13}C,^{15}N-TEDOR-^{13}C,^{13}C-PDSD spectra. a, A,C^{lab}-RNA, **b,** A,U^{lab}-RNA, **c,** G,C^{lab}-RNA, **d,** G,U^{lab}-RNA, **e,** C,U^{lab}-RNA and **f,** A,G^{lab}-RNA at 700 ms (left) and 100 ms (right) PDSD mixing times. Intra- and inter-nucleotide correlations are indicated in green and red, respectively. Overlapped resonances of guanosines, cytidines and adenosines are labelled as G*, C* and A*. Not assigned resonances in (f) represent overlaps of adenosines and guanosines. The total acquisition time was typically 2 days for 2D spectra at 100 ms mixing time and 4.5 days for 2D spectra at 700 ms mixing time.

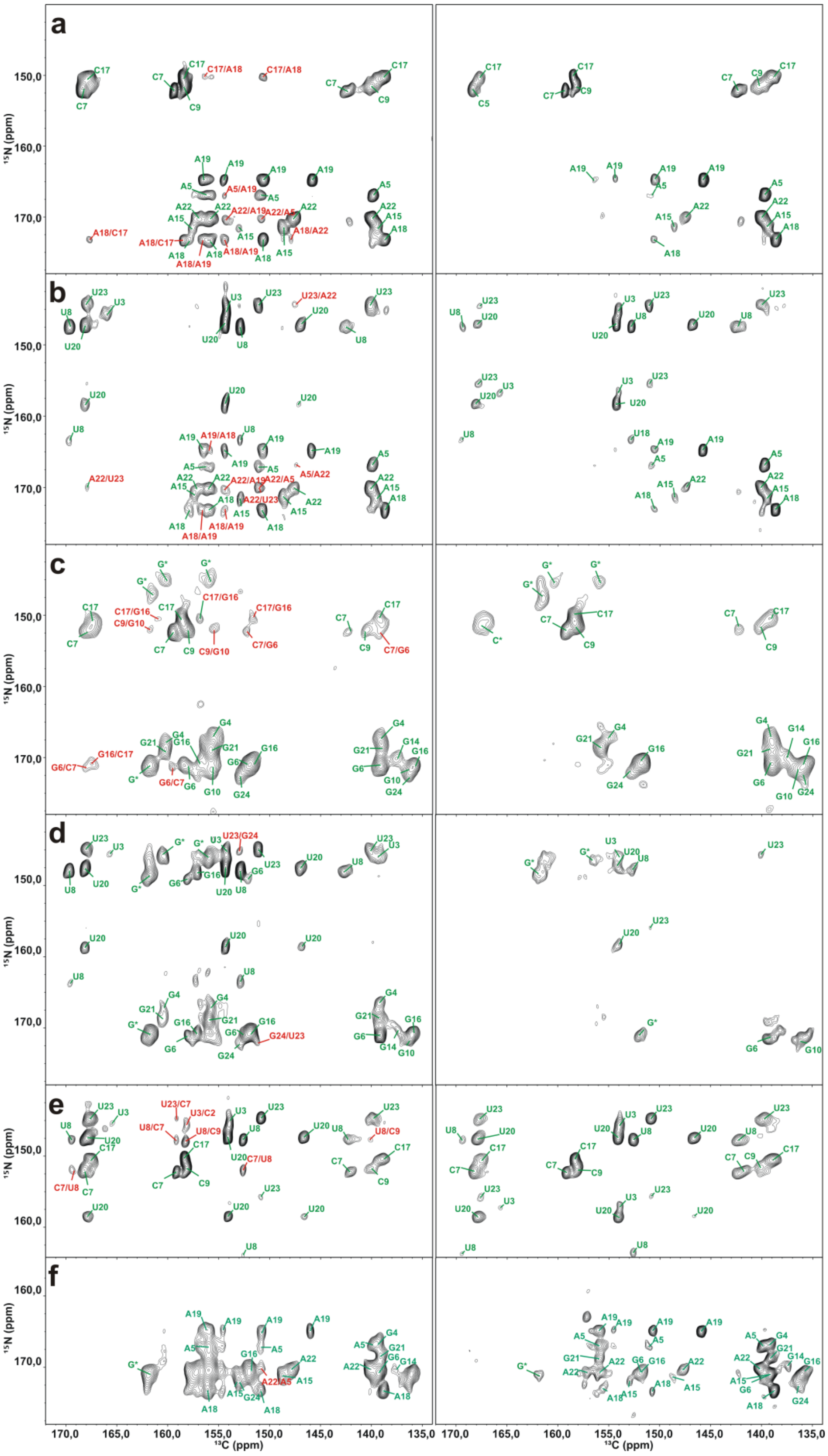

Supplementary Figure 5 | Base regions of 2D $^{13}C,^{15}N$-TEDOR-$^{13}C,^{13}C$-PDSD spectra. a, A,C^{lab}-RNA, **b,** A,U^{lab}-RNA, **c,** G,C^{lab}-RNA, **d,** G,U^{lab}-RNA, **e,** C,U^{lab}-RNA and **f,** A,G^{lab}-RNA at 700 ms (left) and 100 ms (right) PDSD mixing times. Intra- and inter-nucleotide correlations are indicated in green and red, respectively. Overlapped resonances of guanosines, cytidines and adenosines are labelled as G*, C* and A*. Not assigned resonances in (f) represent overlaps of adenosines and guanosines.

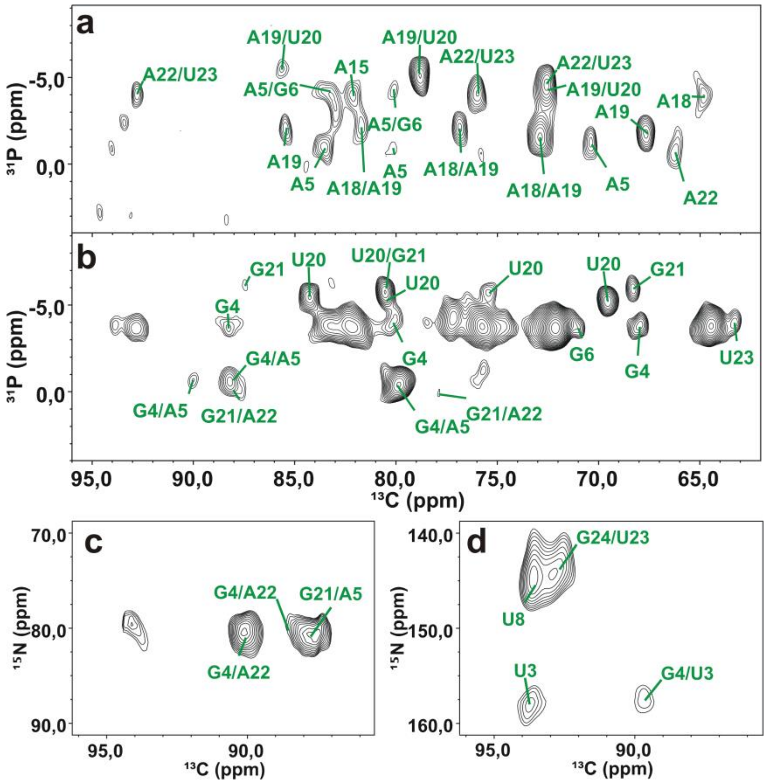

Supplementary Figure 6 | 2D TEDOR spectra. a-b, ^{13}C,^{31}P-TEDOR spectra. **a,** A^{lab}-RNA and **b**, G,U^{lab}-RNA; TEDOR mixing time, 3.2 ms. Non assigned resonances correspond to overlapped nucleotides in helical regions of the RNA. Total acquisition time was 4 days per spectrum. **c-d**, ^{13}C-band-selective,^{15}N-TEDOR spectra. **c,** (G-^{13}C/A-^{15}N)^{lab}-RNA and **d,** (G-^{13}C/U-^{15}N)^{lab}-RNA (mixing time, 12 ms). The total acquisition time was 5 days per spectrum.

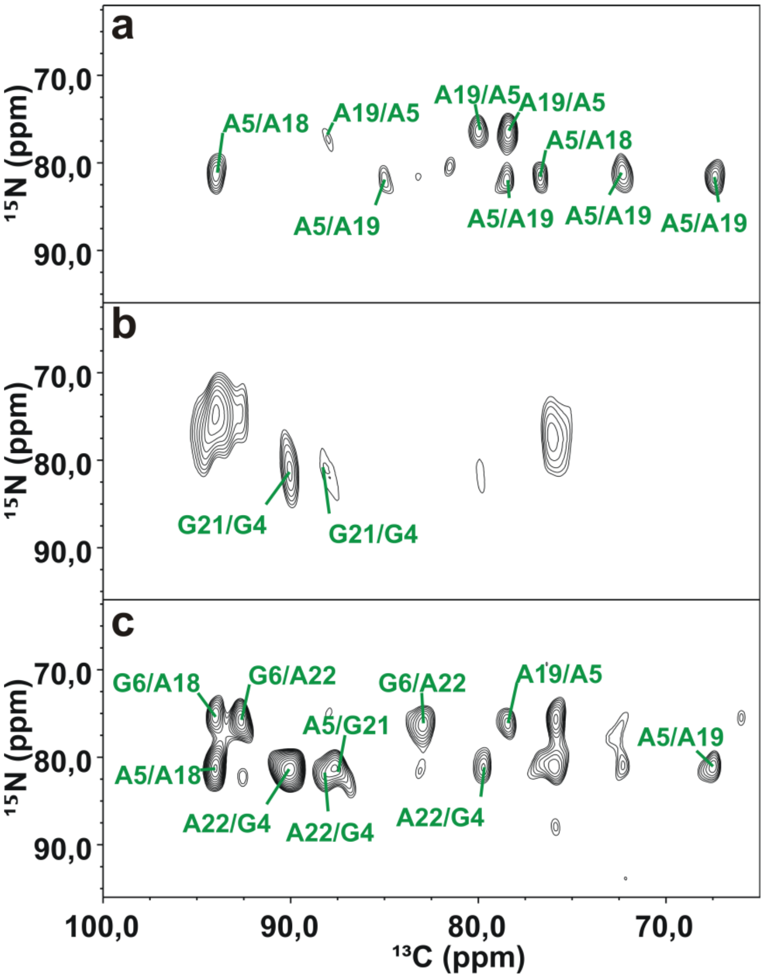

Supplementary Figure 7 | NHHC spectra with 200 µs mixing time. a, A,C^{lab}-RNA, **b,** G,U^{lab}-RNA and **c,** A,G^{lab}-RNA. Total acquisition time was 2 days per spectrum for (a) and (c) and 4 days for (b). Not assigned resonances represent non-resolved overlaps. The resolution of the ^{15}N dimension for the spectrum in (b) was 2.4 times worse than in (a,c).

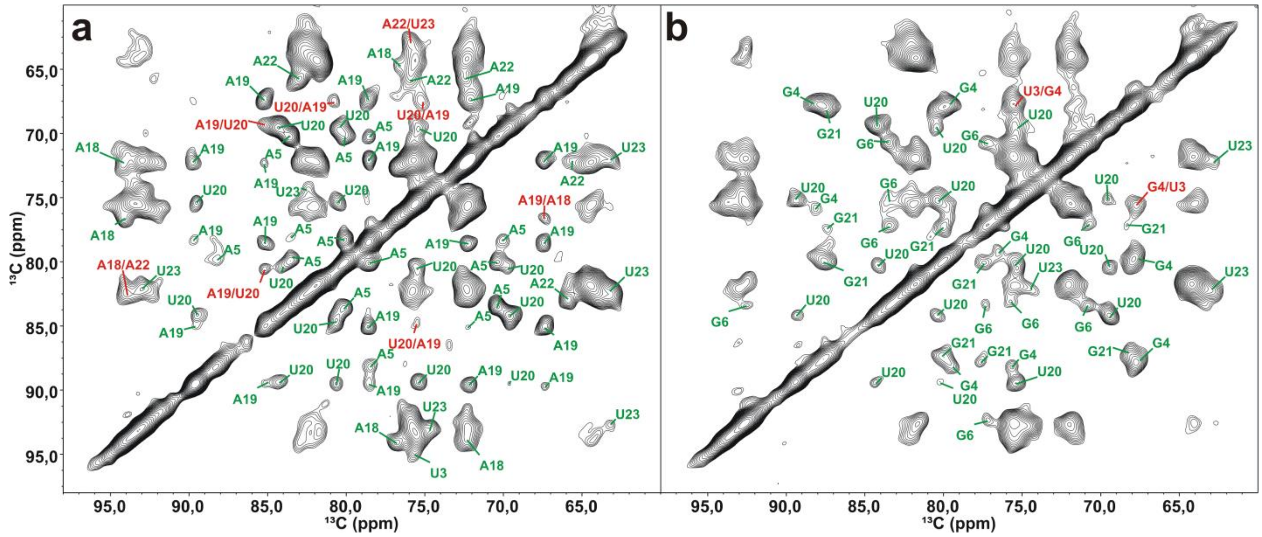

Supplementary Figure 8 | Ribose regions of CHHC spectra with 200 μs mixing time. a, A,U^{lab}-RNA, and **b,** G,U^{lab}-RNA. Intra- and inter-nucleotide correlations are indicated in green and red, respectively. Non-assigned resonances correspond to overlapped peaks. The total acquisition time was 2 days per spectrum.

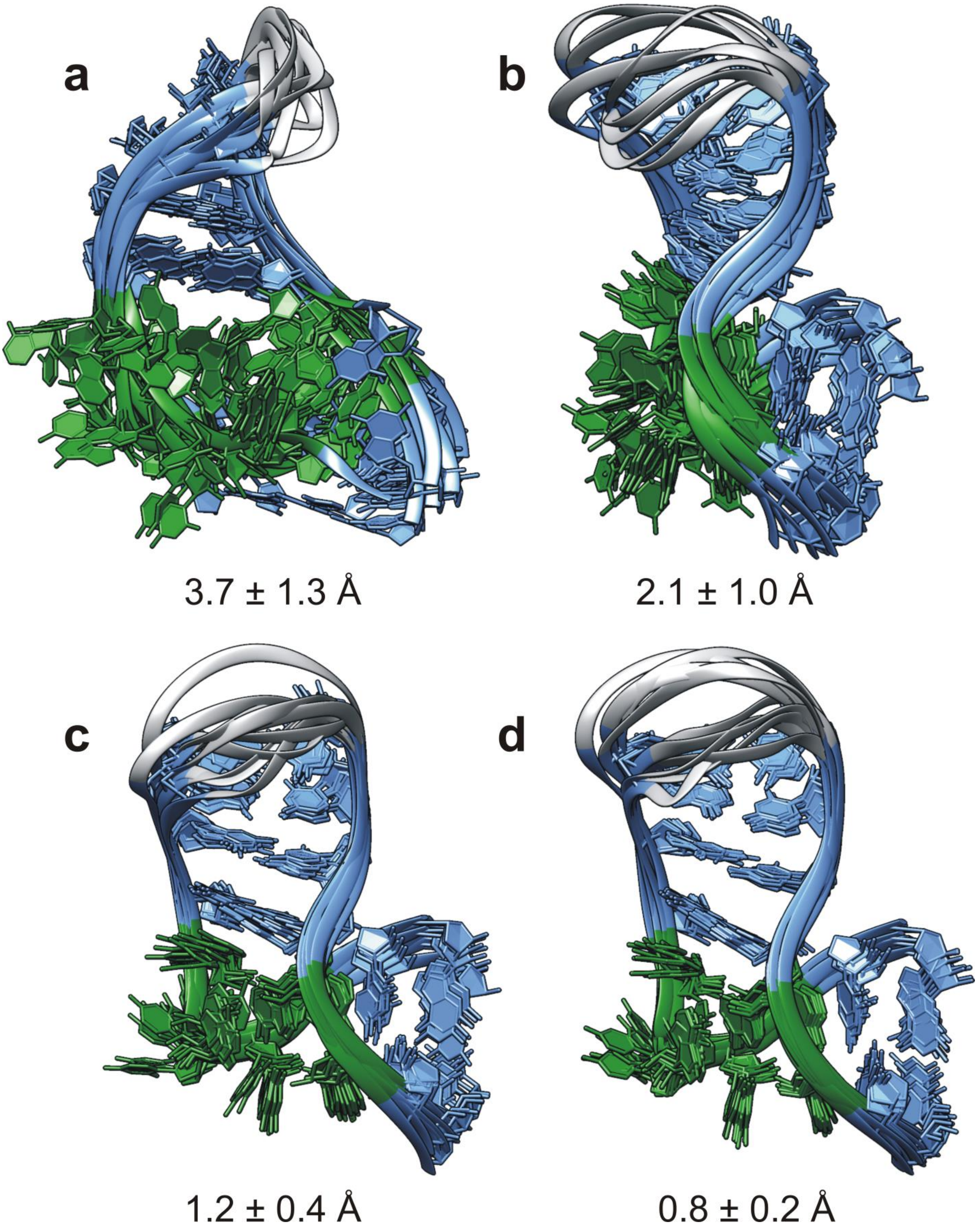

Supplementary Figure 9 | Impact of different restraint classes on the precision of the ssNMR structure. a, Dihedral angles restraints and base-pairs restraints only; **b,** as in (a) with the addition of restraints derived from the ^{13}C,^{15}N-TEDOR-^{13}C,^{13}C-PDSD spectra; **c,** as in (b) with the addition of restraints derived from the CHHC and NHHC spectra; **d,** all restraints. Terminal nucleotides 1 and 25-26 are not shown. Helical regions, light blue; k-turn, green; loop, gray. The precision of the first 20 structures is indicated in each panel as the average backbone r.m.s.d. to the mean structure for residues 2-9 and 14-24, excluding the disordered loop and terminal nucleotides.

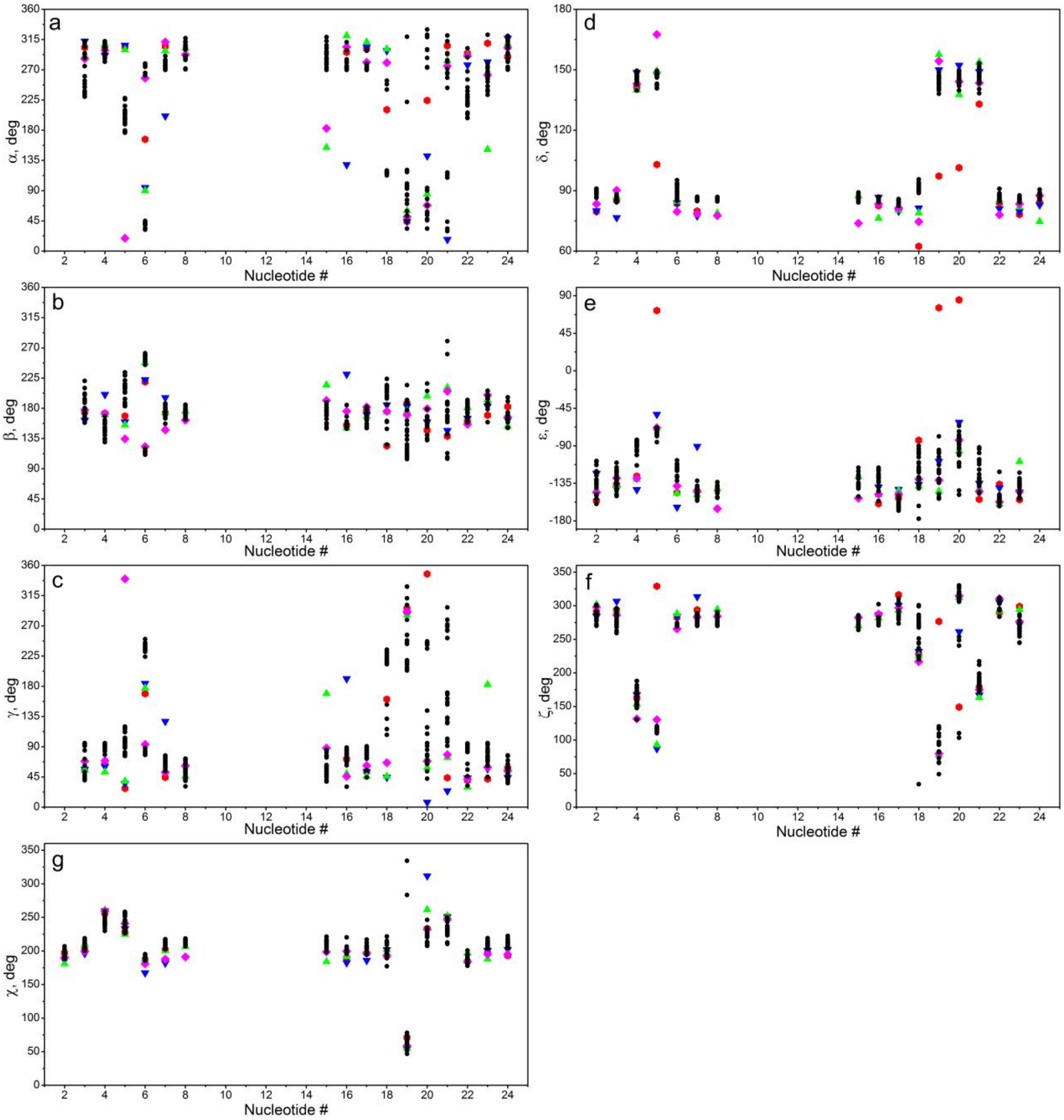

Supplementary Figure 10 | Comparison of ribose dihedrals angles for the 20 lowest energy ssNMR structures (black dots) and orthologous X-ray structures. 1RLG[6] (red hexagons), 3NMU[7] (blue triangles), 4BW0[8] (green triangles), 3PLA[9] (magenta diamonds). **a,** α; **b,** β; **c,** γ, **d,** δ; **e,** ε; **f,** ζ, **g,** χ.

Supplementary Table 1 | Isotropic chemical shifts of the *Pf* 26mer Box C/D RNA in the L7Ae-Box C/D RNA complex.

	Isotropic chemical shifts, ppm																	
	**C1'**	**C2'**	**C3'**	**C4'**	**C5'**	**C2**	**C4**	**C5**	**C6**	**C8**	**N1**	**N2**	**N3**	**N4**	**N6**	**N7**	**N9**	**P**
**C2**	-	-	-	-	-	158.1	-	-	-		-		-	-				-
**U3**	94.8	75.6	71.9	82.9	64.0	154.1	165.6	104.7	*139.3*		145.5		157.1					-
**G4**	90.1	76.0	79.6	88.3	67.8	156.6	155.3	116.7	160.3	139.0	145.0	81.4	-			-	166.9	-3.8
**A5**	88.1	78.4	80.0	83.4	70.3	*156.0*	150.9	118.8	*156.6*	139.8	-		-		81.4	-	166.9	-0.7
**G6**	92.7	75.8	77.3	83.4	70.9	158.2	152.2	119.5	157.9	139	148.9	75.8	-			-	171.1	-3.8
**C7**	93.9	75.4	72.1	82.0	64.3	159.3	168.1	97.1	142.2		152.2		*196.3*	100.0				-
**U8**	93.7	75.5	72.1	82.1	64.3	152.7	169.4	103.2	142.3		147.7		163.6					-
**C9**	93.2	75.9	71.4	*81.2*	64.2	158.2	*168.1*	98.2	140.2		151.7		*197.7*	99.1				-
**G10**	94.0	75.7	71.5	81.8	64.4	-	155.5	-	161.7	136.3	146.2	*75.5*	-			-	171.3	-
**G14**	92.6	74.6	-	-	-	155.9	151.9	*119.2*	-	137.4	147.8	75.4	-			-	170	-
**A15**	92.9	75.6	72.3	81.8	64.1	*153.0*	148.6	*120.6*	*157.6*	139.5	*222.1*		-		-	-	171.5	-
**G16**	92.5	*76.0*	*72.0*	81.7	*64.4*	157.0	151.6	119.0	*160.9*	135.6	147.6	74.6	-			-	170.9	-
**C17**	93.6	75.7	71.7	81.6	64.1	158.5	167.6	97.9	139.0		150.5		*195.9*	99.0				-
**A18**	94.1	76.6	72.5	81.6	64.6	156.0	150.6	121.6	157.7	138.6	-		-		80.7	*230.9*	173.2	-4.2
**A19**	89.7	72.2	78.5	85.1	67.4	154.5	150.5	121.8	156.4	145.8	-		-		76.3	*228.2*	164.7	-1.8
**U20**	89.4	75.4	80.5	84.2	69.5	154.2	167.9	105.7	146.8		147.4		158.5					-5.3
**G21**	87.5	77.5	80.0	87.5	68.3	-	155.7	117.6	160.3	139.0	145.6	81.0	-			-	168.8	-6.0
**A22**	92.4	75.7	72.4	82.9	65.8	155.9	147.5	121.0	157.1	140.0	-		-		81.2	-	170.2	-0.1
**U23**	93.0	74.6	72.0	82.3	63.1	151	167.6	103.8	140.0		144.7		155.7					-4.2
**G24**	92.6	-	-	-	-	*157.4*	152.8	*118.1*	-	136.2	-	76.5	-			-	172.4	-

Assignments derived from only one source and/or from overlapped spectral regions are shown in italic.

Supplementary Table 2 | List of distance restraints used in the structure calculation.

Atom 1		Atom 2		Restrained distance range (Å)	Distance in the ssNMR structure (Å)
		$^{13}C,^{15}N$ TEDOR-$^{13}C,^{13}C$ PDSD			
C17	C1'	G16	C4	3.5-9.0	5.8
C17	C1'	G16	C6	3.5-9.0	6.7
C17	C1'	G16	C2	3.5-9.0	5.8
C17	C1'	G16	C8	3.5-9.0	6.7
C17	C1'	G16	C1'	3.5-9.0	6.1
C7	C1'	G6	C4'	3.5-9.0	7.2
C7	C1'	G6	C3'	3.5-9.0	5.7
C7	C1'	G6	C5'	3.5-9.0	8
C7	C1'	G6	C1'	3.5-9.0	6.8
C7	C1'	G6	C8	3.5-9.0	7.5
C7	C1'	G6	C4	3.5-9.0	6.4
G6	C1'	C7	C2	3.5-9.0	6.9
G6	C1'	C7	C4	3.5-9.0	6.3
G6	C1'	C7	C5	3.5-9.0	5.6
G16	C1'	C17	C4	3.5-9.0	6.1
C9	C1'	G10	C4	3.5-9.0	6.7
C9	C1'	G10	C6	3.5-9.0	6.4
U20	C1'	A19	C3'	3.5-9.0	6.5
U20	C1'	A19	C2'	3.5-9.0	8.1
U20	C1'	A19	C4'	3.5-9.0	6.4
U20	C1'	A19	C5'	3.5-9.0	6.6
A19	C1'	U20	C2'	3.5-9.0	7.9
A19	C1'	U20	C3'	3.5-9.0	7.1
A19	C1'	U20	C4'	3.5-9.0	6.5
A19	C1'	U20	C5'	3.5-9.0	5.2
A19	C1'	A18	C1'	3.5-9.0	8
A19	C1'	A18	C2'	3.5-9.0	6.8
A18	C1'	A19	C4'	3.5-9.0	6.9
A18	C1'	A19	C3'	3.5-9.0	7.6
A18	C1'	A19	C5'	3.5-9.0	5.4
A18	C1'	A19	C2	3.5-9.0	5.5
A18	C1'	A22	C5'	3.5-9.0	6
A18	C1'	A22	C4'	3.5-9.0	5.7
A18	C1'	A22	C4	3.5-9.0	8.5
A5	C1'	A22	C4	3.5-9.0	5.4
A5	C1'	A22	C5	3.5-9.0	5.7
U23	C1'	A22	C5'	3.5-9.0	7.9
U23	C1'	A22	C4	3.5-9.0	4.8
U23	C1'	A22	C2	3.5-9.0	4.3
U23	C1'	A22	C6	3.5-9.0	5.3
U8	C1'	A15	C4	3.5-9.0	8.6
A22	C1'	U23	C2'	3.5-9.0	7.1
A22	C1'	U23	C4	3.5-9.0	6.4
A22	C1'	U23	C2	3.5-9.0	6.5
A22	C1'	A5	C2'	3.5-9.0	7.9

A22	C1'	A5	C3'	3.5-9.0	8.5
A22	C1'	A18	C2'	3.5-9.0	5.8
A22	C1'	A19	C2	3.5-9.0	7.4
A22	C1'	A5	C4	3.5-9.0	4.9
U3	C1'	G4	C5'	3.5-9.0	5.5
U3	C1'	G4	C3'	3.5-9.0	6.9
U3	C1'	G4	C1'	3.5-9.0	5.6
U20	C1'	G21	C4'	3.5-9.0	6.7
U20	C1'	G21	C5'	3.5-9.0	5.9
G21	C1'	U20	C5'	3.5-9.0	6.2
G21	C1'	U20	C4'	3.5-9.0	6.4
G21	C1'	U20	C1'	3.5-9.0	8.3
G24	C1'	U23	C2'	3.5-9.0	4.8
G24	C1'	U23	C2	3.5-9.0	5.8
U23	C1'	G24	C4	3.5-9.0	5.8
G4	C1'	U3	C3'	3.5-9.0	5
G4	C1'	U3	C4'	3.5-9.0	6.5
G4	C1'	U3	C5'	3.5-9.0	7.4
U8	C1'	C7	C2	3.5-9.0	5.8
U8	C1'	C9	C2	3.5-9.0	5.9
U3	C1'	C2	C2	3.5-9.0	5.8
U23	C1'	C7	C2	3.5-9.0	9.7
C7	C1'	U23	C5'	3.5-9.0	5.7
C9	C1'	U8	C2	3.5-9.0	5.6
C9	C1'	U8	C4	3.5-9.0	6.9
C9	C1'	U8	C5	3.5-9.0	7
U8	C1'	C9	C5	3.5-9.0	4.9
U8	C1'	C9	C6	3.5-9.0	4.7
U8	C1'	C7	C5	3.5-9.0	7.2
C2	C1'	U3	C4	3.5-9.0	5.7
C2	C1'	U3	C2	3.5-9.0	6
C2	C1'	U3	C5	3.5-9.0	5
C17	C1'	A18	C2'	3.5-9.0	6.4
C17	C1'	A18	C3'	3.5-9.0	6.4
C17	C1'	A18	C4	3.5-9.0	6.7
A18	C1'	C17	C2	3.5-9.0	4.2
A18	C1'	C17	C4	3.5-9.0	5.2
C17	C1'	A18	C6	3.5-9.0	8.4
C17	C1'	A18	C2	3.5-9.0	8.3
A19	C1'	G21	C1'	3.5-9.0	5.5
A19	C1'	G21	C3'	3.5-9.0	7.4
G6	C1'	A5	C1'	3.5-9.0	3.9
G6	C1'	A5	C2'	3.5-9.0	5
G6	C1'	A5	C3'	3.5-9.0	5
A5	C1'	G6	C2'	3.5-9.0	5
A18	C1'	G21	C3'	3.5-9.0	8.7
			$^{13}C,^{31}P$ TEDOR		
A19	C4'	U20	P	2.0-5.0	3.7

A5	C3'	A5	P	2.0-5.0	4.5
A5	C3'	G6	P	2.0-5.0	2.6
A5	C4'	G6	P	2.0-5.0	3.3
A22	C1'	U23	P	2.0-5.0	4.8
A5	C4'	A5	P	2.0-5.0	3.8
A19	C4'	A19	P	2.0-5.0	3.8
A18	C4'	A19	P	2.0-5.0	3.7
A18	C2'	A19	P	2.0-5.0	3.8
A22	C2'	U23	P	2.0-5.0	3.5
G4	C1'	A5	P	2.0-5.0	4.5
G4	C4'	A5	P	2.0-5.0	3.4
G4	C4'	G4	P	2.0-5.0	3.9
G4	C3'	G4	P	2.0-5.0	4.9
G21	C4'	G21	P	2.0-5.0	3.9
G21	C4'	A22	P	2.0-5.0	3.6
U20	C3'	U20	P	2.0-5.0	5.2
			$^{13}C,^{15}N$ TEDOR		
G4	C1'	A22	N6	3.0-5.0	3.4
G4	C4'	A22	N6	3.0-5.0	4.5
G21	C1'	A5	N6	3.0-5.0	3.9
G6	C1'	A5	N9	3.0-5.0	4.6
G24	C1'	U23	N1	3.0-7.0	6.1
G4	C1'	U3	N1	3.0-7.0	5.4
			CHHC/NHHC		
A18	H2'	A19	H5'	2.0-4.0	3.5
A19	H5'	U20	H3'	2.0-5.0	4.9
A22	H1'	A5	H2	2.0-5.0	3.2
A18	H1'	A22	H4'	2.0-5.0	4.5
A19	H4'	U20	H2'	2.0-5.0	4.4
A19	H4'	U20	H3'	2.0-4.0	3.4
A19	H4'	U20	H5'	2.0-4.0	1.8
A19	H5'	U20	H2'	2.0-5.0	4.7
A22	H2'	U23	H5''	2.0-5.0	4.2
G4	H5'	U3	H2'	2.0-4.0	3.6
G4	H22	G21	H1'	2.0-5.0	5.1
G21	H22	G4	H1'	2.0-5.0	4.8
A19	H62	A5	H2'	2.0-4.0	3.6
A19	H62	A5	H3'	2.0-5.0	5.3
A19	H62	A5	H8	2.0-5.0	4.6
A19	H5''	A5	H61	2.0-4.0	4.9
A19	H4'	A5	H61	2.0-5.0	5.8
A5	H62	A18	H2'	2.0-5.0	6.3
A5	H62	A19	H2'	2.0-5.0	6.2
A5	H61	G21	H1'	2.0-4.0	2.8
A22	H61	G4	H1'	2.0-4.0	2.2
G6	H21	A18	H1'	2.0-4.0	3.8
G6	H22	A22	H4'	2.0-4.0	3.8
G6	H22	A22	H1'	2.0-4.0	4.8

G21	H21	G4	H3'	2.0-5.0	5.8
A22	H61	G4	H3'	2.0-5.0	6
A22	H62	G4	H2'	2.0-5.0	5.1
			Intra-nucleotide		
A19	C4'	A19	C8	2.0-6.0	4.7
A19	C4'	A19	C4	2.0-6.0	4.1
A19	C5'	A19	C8	2.0-6.0	5.5
A19	C5'	A19	C4	2.0-6.0	4.3
A19	C5'	A19	C1'	2.0-4.0	3.4
A19	C5'	A19	C2'	2.0-4.0	3.3
A19	H1'	A19	H3'	2.0-5.0	3.8
A19	H1'	A19	H4'	2.0-5.0	3.4
A19	H1'	A19	H5'	2.0-5.0	4.7
A19	H1'	A19	H8	2.0-4.0	2.5
A19	H2'	A19	H4'	2.0-5.0	3.8
A19	H2'	A19	H5'	2.0-5.0	3.1
A19	H3'	A19	H5'	2.0-5.0	2.2
A18	C5'	A18	C4	2.0-6.0	5.3
A18	C4'	A18	C4	2.0-6.0	4.6
A18	C5'	A18	C1'	2.0-4.0	3.5
A18	C5'	A18	C2'	2.0-4.0	3.7
A18	H1'	A18	H3'	2.0-5.0	3.9
A18	H1'	A18	H4'	2.0-5.0	3.6
A18	H2'	A18	H4'	2.0-5.0	3.9
A18	H2'	A18	H5'	2.0-5.0	4.5
A18	H3'	A18	H5'	2.0-5.0	2.3
A18	H4'	A18	H8	2.0-5.0	4.1
A5	C5'	A5	C4	2.0-6.0	5.5
A5	C5'	A5	C1'	2.0-4.0	3.4
A5	C5'	A5	C2'	2.0-4.0	3.3
A5	H1'	A5	H3'	2.0-5.0	3.8
A5	H2'	A5	H4'	2.0-5.0	3.8
A5	H2'	A5	H5'	2.0-5.0	4.3
A5	H2'	A5	H8	2.0-5.0	2.4
A5	H3'	A5	H5'	2.0-5.0	3.5
A22	C5'	A22	C4	2.0-6.0	5.5
A22	C3'	A22	C4	2.0-6.0	4.2
A22	C5'	A22	C1'	2.0-4.0	3.6
A22	C5'	A22	C2'	2.0-4.0	3.7
A22	H4'	A22	H8	2.0-5.0	4.3
A22	H2'	A22	H5'	2.0-5.0	5.4
A22	H3'	A22	H5'	2.0-5.0	3.7
A15	C5'	A15	C1'	2.0-4.0	3.6
A15	C5'	A15	C2'	2.0-4.0	3.7
U3	C5'	U3	C1'	2.0-4.0	3.6
U3	C5'	U3	C2'	2.0-4.0	3.7
U3	C5'	U3	C2	2.0-6.0	5.6
U8	C5'	U8	C1'	2.0-4.0	3.6

U8	C5'	U8	C2'	2.0-4.0	3.7
U8	C5'	U8	C2	2.0-6.0	5.6
U20	C5'	U20	C1'	2.0-4.0	3.3
U20	C5'	U20	C2'	2.0-4.0	3.2
U20	C5'	U20	C2	2.0-6.0	5.4
U20	C5'	U20	C6	2.0-6.0	3.8
U23	C5'	U23	C1'	2.0-4.0	3.6
U23	C5'	U23	C2'	2.0-4.0	3.7
U23	C5'	U23	C2	2.0-6.0	5.6
G4	C5'	G4	C1'	2.0-4.0	3.4
G4	C5'	G4	C2'	2.0-4.0	3.3
G6	C5'	G6	C1'	2.0-4.0	3.5
G6	C5'	G6	C2'	2.0-4.0	3.7
G10	C5'	G10	C1'	2.0-4.0	3.5
G10	C5'	G10	C2'	2.0-4.0	3.7
G21	C5'	G21	C1'	2.0-4.0	3.5
G21	C5'	G21	C2'	2.0-4.0	3.4
C7	C5'	C7	C1'	2.0-4.0	3.6
C7	C5'	C7	C2'	2.0-4.0	3.7
C9	C5'	C9	C1'	2.0-4.0	3.6
C9	C5'	C9	C2'	2.0-4.0	3.7
C17	C5'	C17	C1'	2.0-4.0	3.6
C17	C5'	C17	C2'	2.0-4.0	3.7

Supplementary references

1. Cherepanov, A. V., Glaubitz, C., & Schwalbe, H. High-resolution studies of uniformly C-13,N-15-labeled RNA by solid-state NMR spectroscopy. *Angew. Chem. Int. Ed.* **49,** 4747-4750 (2010).

2. Jaroniec, C. P., Filip, C., & Griffin, R. G. 3D TEDOR NMR experiments for the simultaneous measurement of multiple carbon-nitrogen distances in uniformly C-13, N-15-labeled solids. *J. Am. Chem. Soc.* **124,** 10728-10742 (2002).

3. Bennett, A. E., Ok, J. H., Griffin, R. G., & Vega, S. Chemical-shift correlation spectroscopy in rotating solids - Radio Frequency-driven Dipolar Recoupling and longitudinal exchange. *J. Chem. Phys.* **96,** 8624-8627 (1992).

4. Lange, A., Luca, S., & Baldus, M. Structural constraints from proton-mediated rare-spin correlation spectroscopy in rotating solids. *J. Am. Chem. Soc.* **124,** 9704-9705 (2002).

5. Lange, A., *et al.* A concept for rapid protein-structure determination by solid-state NMR spectroscopy. *Angew. Chem. Int. Ed.* **44,** 2089-2092 (2005).

6. Moore, T., Zhang, Y. M., Fenley, M. O., & Li, H. Molecular basis of box C/D RNA-protein interactions: Cocrystal structure of archaeal L7Ae and a box C/D RNA. *Structure* **12,** 807-818 (2004).

7. Xue, S., *et al.* Structural basis for substrate placement by an archaeal Box C/D ribonucleoprotein particle. *Mol. Cell* **39,** 939-949 (2010).

8. Huang, L. & Lilley, D. M. The molecular recognition of kink-turn structure by the L7Ae class of proteins. *RNA* **19,** 1703-1710 (2013).

9. Lin, J., *et al.* Structural basis for site-specific ribose methylation by box C/D RNA protein complexes. *Nature* **469,** 559-563 (2011).
